# UNUSUAL EXTERNAL BLOCKAGE - NECK ABSCESS LEADING TO EXTERNAL JUGULAR VEIN THROMBOSIS: AN UNCOMMON COMPLICATION

**DOI:** 10.51894/001c.122826

**Published:** 2024-08-30

**Authors:** Shamaiza Waqas, Khin Pyai, Waqas Abid

**Affiliations:** 1 Internal Medicine Ascension Macomb Oakland Hospital

## 14

### BACKGROUND

External jugular vein thrombosis is a rare condition that often goes unnoticed. Previous cases have been attributed to causes such as central venous catheterization, head and neck interventions, malignancy, IV drug abuse, trauma-related incidents, and idiopathic factors. Other potential causes include increasing age, obesity, underlying illnesses, and external compression. Here, we present a case of external jugular vein thrombosis associated with anterior neck abscess resulting from intravenous drug use.

### CASE DESCRIPTION

The patient was a 22-year-old male with a past medical history of IV drug use disorder. He presented with complaints of right-sided neck pain and swelling. On examination, the patient had a right-sided anterior neck mass with surrounding skin erythema, warmth and tender to touch. Blood pressure 149/94 mmHg, heart rate 139 bpm, and temperature 101.7°F. The patient reported IV drug use in the right side of his neck before the onset of these symptoms. His blood work showed WBC 20.7, ESR 117 mm/h, CRP 161.3 mg/L. His D-dimer was elevated 2050 ng/mL. ASO titers were 1523 IU/mL. Blood cultures revealed group A streptococcus. CT scan with contrast of the soft tissue in the neck showed a large abscess in the right anterior neck and thrombosis of the right external jugular vein. The patient was started on IV antibiotics. Incision and drainage of the right anterior neck abscess was performed. Vascular surgery was consulted, but the patient was not a candidate for anticoagulation as the thrombosis was likely secondary to IV drug use in the anterior neck vessel.

### DISCUSSION

Unlike the usual association of head and neck infections with internal jugular vein thrombosis, our patient exhibited involvement of the external jugular vein. Managing external jugular vein thrombosis remains challenging. CT scans have been utilized for diagnosis; recent studies suggest that ultrasound may be more suitable. Treatment approaches for external jugular vein thrombosis vary depending on the underlying cause. Options include medical management with antibiotics and NSAIDs, surgical removal of the thrombus, or conservative management. Unlike internal jugular vein thrombosis, where anticoagulation is commonly prescribed, anticoagulation in external jugular vein thrombosis should be tailored to the individual patient, considering pre-existing risk factors, comorbidities, and other patient-specific factors.

